# Acute respiratory distress syndrome after aspiration of lamp oil in a fire-eater: a case report

**DOI:** 10.1186/s13256-016-0960-1

**Published:** 2016-07-07

**Authors:** Nico Behnke, Jekaterina Breitkreuz, Cordula Buck, Marc Hinterthaner, Alexander Emmert

**Affiliations:** 1 Department of Thoracic and Cardiovascular Surgery, University of Göttingen, Robert-Koch-Str. 40, D-37075 Göttingen, Germany; 2 Department of Pulmonology, University of Göttingen, Robert-Koch-Str. 40, 37075 Göttingen, Germany; 3 Department of Internal Medicine, University of Göttingen, Robert-Koch-Str. 40, 37075 Göttingen, Germany

**Keywords:** Case report, ARDS, Aspiration, Lamp oil, Fire-eater

## Abstract

**Background:**

Fire-eater’s pneumonia is an exogenous chemical pneumonitis after accidental aspiration of hydrocarbon fluids during the act of fire-eating. There have been few case reports in the literature regarding complications after fire-eating but so far none, to the best of our knowledge, have described such drastic and life-threatening pulmonary complications as in this case while only having swallowed a small amount of fluid.

**Case presentation:**

We present a case of fire-eater’s pneumonia in a 28-year-old white man with severe pulmonary complications. He presented with pneumonitis and partial respiratory insufficiency. He was diagnosed with acute respiratory distress syndrome and was treated with antibiosis, oxygen therapy, and required non-invasive ventilation. He had a good recovery.

**Conclusions:**

Accidental aspiration of even small amounts of lamp oil can lead to serious life-threatening pulmonary complications. Although fire-eaters are a comparatively small occupational group, the severity of possible complications illustrates that awareness of these consequences should be raised in teenagers and young adults who might be tempted into trying it. This case in a Western country shows that the dangers of fire-eating are not to be underestimated and are not limited to Eastern European countries where the majority of cases have been reported.

## Background

Fire-eater’s lung is an exogenous chemical pneumonitis after accidental aspiration of hydrocarbon fluids during the act of fire-eating. Classic symptoms include dyspnea, cough, high fever, severe chest pain, hemoptysis, elevated inflammatory markers a few hours after aspiration, unilateral or bilateral alveolar infiltrates, lower lobe involvement, pleural effusion, and pneumatoceles [1–7] with initial signs only a few hours after inhalation [8].

Although acute symptoms may be life-threatening, patients usually show a good recovery [2, 4].

Fire-eating is relatively uncommon making fire-eater’s pneumonia a rare condition for which, to the best of our knowledge, a guideline has not yet been established [7].

## Case presentation (Table 1)

A 28-year-old white man was admitted to our intensive care unit from another hospital, 2 days after admission following accidental aspiration of ¼ mouthful of lamp oil during a fire-eating performance. Up to this point he had been diagnosed with pneumonitis, partial respiratory insufficiency and required non-invasive ventilation. In addition, he had had infiltrative alterations in the lower lobe of his right lung, diaphragmatic elevation on the right side and pleural thickening with effusion.

**Table 1:** Timeline of case

Aspiration of lamp oil	11 July 2015
Admission to first hospital	12 July 2015
Referral to University of Göttingen	14 July 2015
Discharge from University of Göttingen	06 August 2015

After admission to our intensive care unit he was stable with an oxygen flow of 6L. He complained of chest pain during breathing and moving compatible with pleuritis. During the night he developed tachypnea and tachycardia.

**Fig. 1:**
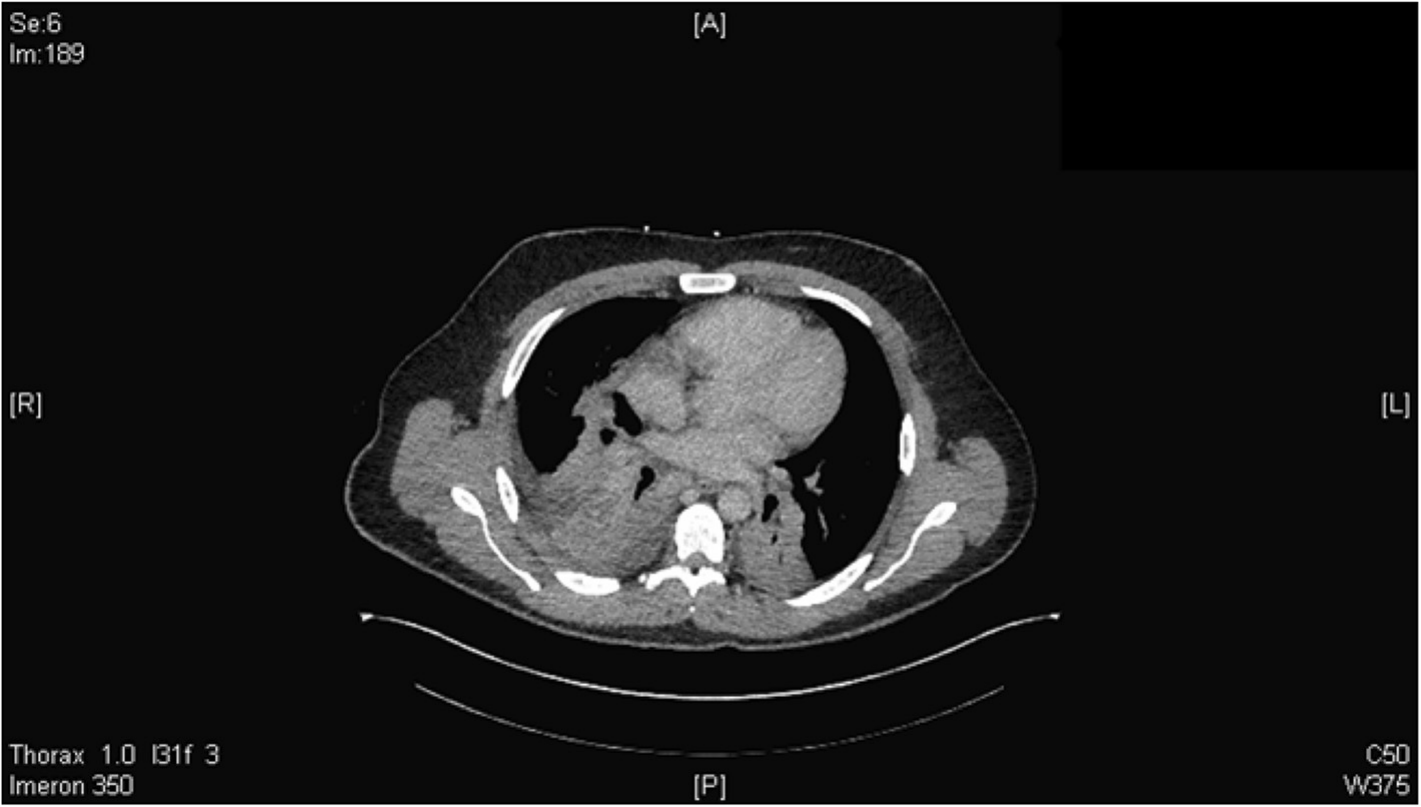
Basal lung infiltrations on both sides. Hypodense inhomogeneous pulmonary pockets with suspected basal infiltrations on both sides or initial abscess formation as differential diagnosis

**Fig. 2:**
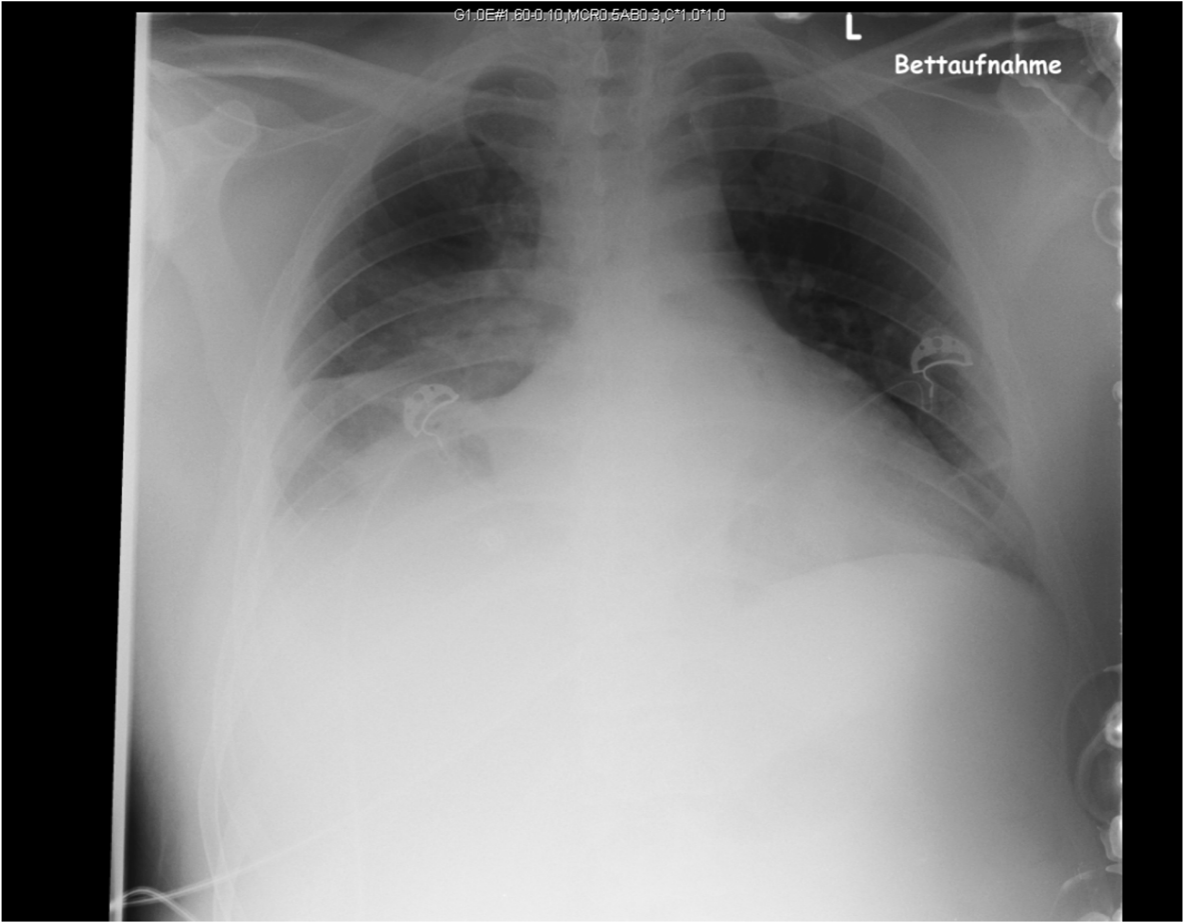
Extensive and patchy basal lung infiltrations on both sides. Extensive and patchy basal lung infiltrations on both sides with more infiltrations on the right than the left side and possible pleural effusion as differential diagnosis, image taken in bed

Because of suspected superinfection and C-reactive protein (CRP) as high as 404 mg/L we drew blood cultures, which later turned out to be negative, and changed antibiosis from sultamicillin to tazobactam and metronidazole. To improve oxygenation we changed to intermittent AirVo therapy (humidifier with integrated flow generator). A computed tomography scan of his chest showed hypodense inhomogeneous pulmonary pockets with suspected basal infiltrations on both sides or initial abscess formation as differential diagnosis (Fig. 1). A chest X-ray examination showed extensive and patchy basal lung infiltrations on both sides with more infiltrations on the right than the left side and possible pleural effusion as differential diagnosis (Fig. 2). The mediastinal window showed a lymphadenopathy and enlarged hilar lymph nodes on this right side.

Under antipyretic drug treatment and oxygen therapy, he started to feel better and mobilization to a chair and small steps were possible. Three days after admission to our intensive care unit he was transferred to a regular ward. After intensive physical and breathing training his general condition improved, he needed less oxygen and his inflammatory markers decreased. A chest X-ray showed progressive opacity in the right lower area in accordance with pleural effusion, which was treated by drainage placement after sonographic verification. A microbiological analysis showed no pathogens.

**Fig. 3:**
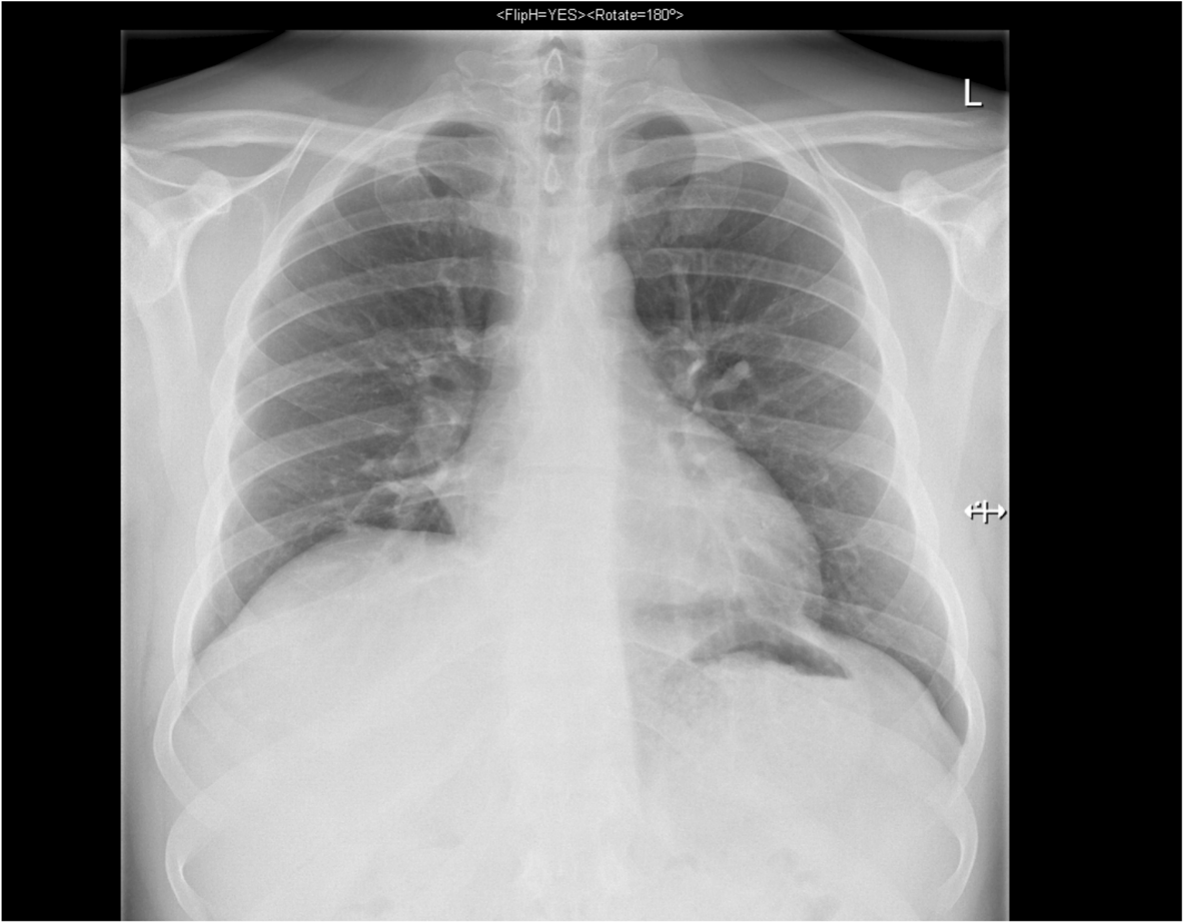
Regressive dystelectatic alterations in both lower lobes. In comparison to earlier imaging this image shows regressive dystelectatic alterations; no new infiltrations, pneumothorax or pleural effusion

He then developed high fever with right cervical lymph node enlargement after which another set of blood cultures was drawn which again showed no pathogens. After switching to meropenem his symptoms decreased. By the time he was discharged (27 days after aspiration event) a chest X-ray showed regressive dystelectatic alterations in both lower lobes (Fig. 3). His pulmonary function still showed a restrictive ventilation defect.

## Discussion

In 1925 Laughlen published the first cases of documented exogenous pneumonitis in autopsies after three children had been treated orally or nasally with oily substances [9]. He deduced that oil is responsible for consolidations of the lung if it comes into contact with lung tissue. Over time a few case reports of exogenous chemical pneumonitis have been published with the majority of cases due to accidental aspiration during fire-eating. Fire-eating is relatively uncommon making fire-eater’s pneumonia a rare condition for which, to the best of our knowledge, a guideline has not yet been established [7, 10].

Our patient showed symptoms similar to those already documented in the literature. His condition required intensive care and consequent observation of vital signs. In accordance with the literature, a conservative approach was chosen and our patient recovered within 3 weeks to the point of being able to be discharged without the need for oxygen therapy and in a greatly improved general condition. During hospitalization no steroids were given as opposed to other cases [2, 3, 5]. This approach was in line with others [5, 11] who also gave no steroid treatment because it does not improve the outcome [12].

A control X-ray of his chest before discharge still showed dystelectatic alterations in his lower lobes and an enlarged hilus. A *restitutio ad integrum* (restoration to original condition) as others have reported [5, 13] was not observed but it is possible that over time a *restitutio ad integrum* could be reached. It is likely that in light cases even no treatment can lead to complete recovery as was shown by Kadakal *et al.* [14].

## Conclusions

Accidental aspiration of even small amounts of lamp oil can lead to serious life-threatening pulmonary complications. Although fire-eaters are a comparatively small occupational group, the severity of possible complications illustrates that awareness of these consequences should be raised in teenagers and young adults who might be tempted into trying it. This case in a Western country shows that the dangers of fire-eating are not to be underestimated and are not limited to Eastern European countries where the majority of cases have been reported.
